# Linking IL-10 signaling with lipid metabolic programs in macrophages: dysregulated ceramide homeostasis drives colitis

**DOI:** 10.1038/s41392-024-01864-7

**Published:** 2024-06-08

**Authors:** Imke Atreya, Markus F. Neurath

**Affiliations:** 1 Department of Medicine 1, University Hospital of Erlangen, Friedrich-Alexander University Erlangen-Nürnberg, Erlangen, Germany; 2 Deutsches Zentrum Immuntherapie (DZI), Erlangen, Germany

**Keywords:** Gastrointestinal diseases, Inflammation

In a recent study published in *Nature*, York and coworkers identified the immunometabolic regulation of mono-unsaturated fatty acid (MUFA) pools and the subsequent increase in sphingolipid biosynthesis in TLR2-activated macrophages as an important link between impaired IL-10 signaling and the development of intestinal inflammation.^1^ Thus, targeted interference with the newly identified IL-10/MUFA/sphingolipid axis appears as a promising strategy to restore immunometabolic homeostasis in the intestine of patients with inflammatory bowel diseases (IBD).

The critical dependence of gut health on the anti-inflammatory cytokine IL-10 is well established.^2^ To mention some key evidence, knockout (KO) mice lacking IL-10 or one of the IL-10 receptor subunits develop spontaneous colitis, while mutations within the coding region of the human IL-10 or IL-10 receptor genes account for a relevant percentage of patients diagnosed with very early-onset IBD. Although IL-10 can be sensed by various immune cells, a macrophage-restricted loss of the IL-10RA receptor subunit was sufficient to induce spontaneous colitis, implicating mucosal macrophages as central mediators of anti-inflammatory IL-10 signaling.^2^ Despite the well-described colitis-promoting consequence of in vivo IL-10 blockade/neutralization, the intracellular cascade linking IL-10 receptor ligation to the regulation of inflammatory macrophage function remains incompletely understood. Interestingly, a previous study suggested that the anti-inflammatory function of IL-10 in macrophages involves its ability to block activation-induced metabolic reprogramming, thus describing its cellular effects as an interplay between immunological signaling and metabolic regulation.^3^ From a translational perspective, there is a growing awareness of the relevance of metabolic processes for immune cell function in the context of IBD. This awareness arises in particular from the clinical need to offer optimized treatment strategies to more than 30% of IBD patients who do not show a satisfying response to the currently available therapeutic regimens. New targets that allow modulation of the metabolic response of immune cells to inflammatory triggers, rather than exclusively blocking classical immune mediators upstream or downstream of cytokine receptors, may be particularly beneficial for those IBD patients who do not respond to biological agents such as anti-TNF therapy, vedolizumab, p19/p40 blockers and JAK inhibitors.

Since the lipid pool in macrophages is known to be altered during TLR-mediated activation, York et al.^1^ first focused on whether this reprogramming of lipid metabolism depends on IL-10, whose secretion by macrophages is also induced by TLR ligation. Indeed, the lipid metabolomic data indicated that TLR2-induced IL-10 signaling significantly affects sphingolipid metabolism of macrophages. Based on direct infusion mass spectrometry, TLR2-activated IL-10-deficient macrophages could be characterized by increased levels of ceramides and hexosyl ceramides compared to IL-10-positive control macrophages, whereas all unsaturated and some saturated sphingomyelins were decreased in the absence of IL-10. The accumulation of ceramides in TLR2-activated IL-10 KO macrophages appeared to be driven by increased de novo ceramide synthesis. Accordingly, genetic deletion of ceramide synthase Cers2, an enzyme that controls very long chain (VLC) ceramide synthesis, prevented the induction of inflammatory genes in in vitro activated macrophages by IL-10R blockade and, under in vivo conditions, IL-10 receptor/Cers2 double knockout bone marrow chimeric mice showed milder signs of intestinal inflammation than cohoused IL-10 receptor knockout chimeric mice.

**Fig. 1:**
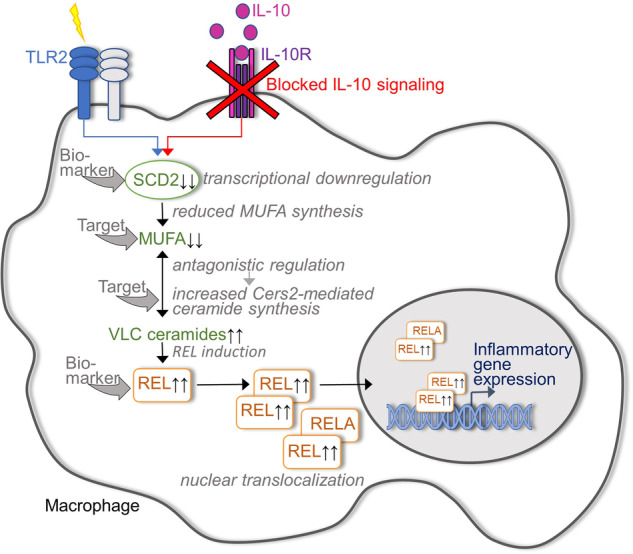
The insufficient capacity of IL-10-deficient macrophages to upregulate MUFA synthesis represents the underlying cause of their pathologically increased inflammatory activity. Hypothesis of the underlying immunometabolic signaling cascade: Blockade of IL-10 signaling in toll-like receptor 2 (TLR2) activated macrophages results in a transcriptional downregulation of the enzyme stearoyl-CoA desaturase 2 (SCD2), which catalysis the synthesis of mono-unsaturated fatty acids (MUFA). The subsequent decrease of cellular MUFA levels causes (via a not yet exactly defined mechanism) an accumulation of very long chain (VLC) ceramides. Here, the enzyme ceramide synthase 2 (Cers2) is relevantly involved in the synthesis of VLC ceramides. Finally, VLC ceramides are able to trigger the expression of inflammatory genes via the induction and nuclear translocation of the NF-kappaB family transcription factor REL. Potential candidate targets for innovative biomarker or therapeutic concepts in IBD are indicated

The next step was to elucidate the cascade underlying the regulation of ceramide biosynthesis and the accumulation of VLC ceramides in IL-10 KO macrophages. Somewhat unexpectedly, the expression levels of all genes directly involved in sphingolipid metabolism turned out to be comparable between IL-10 KO and control macrophages. However, IL-10 deficiency in macrophages caused a significant downregulation of the enzyme stearoyl-CoA desaturase 2 (SCD2), which catalyzes the formation of MUFAs. These findings led to the hypothesis that MUFA synthesis links inflammation and VLC ceramide synthesis in IL-10 KO macrophages. Indeed, stable-isotope tracer analysis indicated that the reduced SCD2 expression in TLR2-stimulated IL-10 KO macrophages was associated with reduced MUFA synthesis, whereas the inflammatory gene expression profile of IL-10 KO macrophages and their increased VLC ceramide biosynthesis could be attenuated by exogenous MUFA supplementation. Furthermore, SCD2-deficient macrophages phenocopied IL-10 KO macrophages in terms of VLC ceramide accumulation and increased inflammatory gene expression. Taken together, York et al. were able to identify the insufficient capacity of IL-10-deficient macrophages to upregulate MUFA synthesis as the underlying cause of their pathologically enhanced inflammatory activity (Fig. 1).

But how does the IL-10-controlled availability of VLC ceramides affect the regulation of inflammatory genes? York et al.^1^ interestingly demonstrated that the NF-kappaB family transcription factor REL is required for the ability of VLC ceramides to induce inflammatory gene expression in TLR2-activated macrophages and, accordingly, for the full development of intestinal inflammation in IL-10-deficient mice. Interestingly, in vitro analyses of activated IL-10/c-Rel double knockout macrophages implicated that the ceramide-induced activation of REL predominantly supports the prolonged maintenance of inflammatory gene expression rather than enhancing its early initiation.

Regarding the therapeutic targetability of IL-10 signaling, previous data from experimental mouse models suggested that direct administration of recombinant IL-10 may ameliorate colitis,^2^ but clinical IBD trials have been disappointing overall. The new insights into the immunometabolic cascade that is initiated when macrophages are activated in the absence of IL-10 signaling may pave the way for more clinically successful strategies to elicit IL-10-triggered anti-inflammatory effects, which may even be beneficial for IBD patients with IL-10 receptor dysfunction. Nutritional MUFA supplementation or targeted blockade of the VLC ceramide synthesis via inhibition of Cers2 appear, at least on the first view, as two attractive strategies in this context (Fig. 1). However, assuming that both these approaches cannot be implemented in a macrophage-restricted way, their expected impact on other cells involved in the maintenance of intestinal homeostasis must be considered. One potential limitation of the study is the lack of validation in other IL-10 receptor-expressing intestinal immune cells, including those of humans. Cers2 represents a tumor metastasis suppressor gene,^4^ and defined MUFAs have been associated with enhanced T effector cell function^5^ underlining potential risk factors when targeting this pathway. Thus, the validation of the findings on the IL-10/MUFA/VLC ceramide/REL axis in other IL-10 receptor-expressing intestinal immune cells, in addition to macrophages, is essential. Moreover, the validation of this axis in human immune cells in the inflamed intestine of IBD patients is of paramount importance.

The more complete the molecular consequences of insufficient IL-10 signaling in innate and adaptive immune cells and in the intestinal epithelium can be deciphered, the better the chances are to fully exploit the high translational potential of this study. In terms of personalized medicine, rationally designed biomarker strategies based on the intestinal expression profile of genes/proteins that regulate MUFA synthesis and/or REL activation could reliably identify individual IBD patients with relevant impaired IL-10 signaling who could best benefit from targeted local corrections of MUFA/VLC ceramide homeostasis.
